# Looking at plant cell cycle from the chromatin window

**DOI:** 10.3389/fpls.2014.00369

**Published:** 2014-07-25

**Authors:** Bénédicte Desvoyes, María Fernández-Marcos, Joana Sequeira-Mendes, Sofía Otero, Zaida Vergara, Crisanto Gutierrez

**Affiliations:** Centro de Biologia Molecular Severo Ochoa, Consejo Superior de Investigaciones Cientificas, Universidad Autónoma de MadridMadrid, Spain

**Keywords:** cell cycle, chromatin, epigenetics, gene expression, DNA replication, *Arabidopsis*, plant

## Abstract

The cell cycle is defined by a series of complex events, finely coordinated through hormonal, developmental and environmental signals, which occur in a unidirectional manner and end up in producing two daughter cells. Accumulating evidence reveals that chromatin is not a static entity throughout the cell cycle. In fact, there are many changes that include nucleosome remodeling, histone modifications, deposition and exchange, among others. Interestingly, it is possible to correlate the occurrence of several of these chromatin-related events with specific processes necessary for cell cycle progression, e.g., licensing of DNA replication origins, the E2F-dependent transcriptional wave in G1, the activation of replication origins in S-phase, the G2-specific transcription of genes required for mitosis or the chromatin packaging occurring in mitosis. Therefore, an emerging view is that chromatin dynamics must be considered as an intrinsic part of cell cycle regulation. In this article, we review the main features of several key chromatin events that occur at defined times throughout the cell cycle and discuss whether they are actually controlling the transit through specific cell cycle stages.

## INTRODUCTION

The cell division cycle is normally divided into phases with a defined temporal order. These are G1, the first phase entered upon completion of cytokinesis where cells commit for a new cell division and prepare for genome duplication, S-phase where chromosome replication occurs, G2, a time for checking genome integrity and preparing for chromosome segregation, and mitosis (M), including cytokinesis, where both the replicated genome and the rest of cytoplasmic components are divided into the two newborn cells. This relatively simplistic view is actually the result of an extraordinarily complex and regulated series of events that lead to the characteristic unidirectionality of cell cycle progression (Gutierrez, 2009). In fact, the many different processes required for successful completion of the cell division cycle are highly coordinated. In the case of plants, with a typical postembryonic and continuous organogenesis, development relies mainly on cell proliferation and endoreplication, hence the cell division potential is developmentally regulated (Gutierrez, 2005).

The two major transitions in cell cycle are the G1/S and G2/M that initiate the genome duplication and segregation phases, respectively. Both of them involve dramatic changes at the chromatin level that were thought to occur in a passive manner as a consequence of cell cycle progression but recent data indicate that they also determine the efficiency of cell cycle transitions. Contrary to the apparently repeated and monotonous organization of eukaryotic chromatin as a string of nucleosomes, it is a highly dynamic entity. In addition to differences in histone composition of each nucleosome unit due to the presence of variants of the canonical histones H2A, H2B, H3, and H4, a large number of residues, particularly at the N-terminal tail of histone H3 can be modified by acetylation, methylation, phosphorylation, ubiquitynation, and citrullination, among others (Kouzarides, 2007). This produces a high combinatorial complexity that, we are learning, is at the basis of chromatin processes such as replication, transcription, recombination, repair, splicing, silencing, chromosome organization, etc. Moreover, nucleosomes can be displaced and rearranged by chromatin remodeling complexes, therefore modifying their position relative to genomic features, e.g., transcriptional start sites, promoters, replication origins, etc.

All these chromatin modifications have direct consequences on the local accessibility of certain DNA regions by cellular factors, e.g., transcription factors (TFs). Thus, histone composition of nucleosomes, their precise location relative to gene features, as well as histone and DNA modifications can have a profound effect on transcriptional patterns (Nelissen et al., 2007; Probst et al., 2009; Ingouff and Berger, 2010; Law and Jacobsen, 2010; Otero et al., 2014). In fact, many of them occur in a cell cycle-dependent manner. An attractive hypothesis is that some histone modifications actually drive certain stages of cell cycle (Sanchez et al., 2008; Gondor and Ohlsson, 2009; Liu et al., 2010; Tardat et al., 2010). This connects directly to another feature associated with cell cycle progression, transcriptional control of many genes. At a higher level of complexity, genome organization within the 3D organization of the nucleus appears to be of primary relevance. Thus, eukaryotic genomes, including plant genomes, are packed and organized in a non-random manner within the nucleus, in such a way that individual loci occupy specific sites in the nucleus (Paul and Ferl, 1998). Furthermore, the physical proximity of genes that can be far away in the linear scale of the chromosome creates chromatin microenvironments that allow or facilitate novel regulatory combinations, increasing the plasticity of cellular response and adaptability (Cao et al., 2014). Genome wide approaches to determine the spatial contacts of individual loci have recently applied in plants (Grob et al., 2013). It would be extremely exciting to decipher how such contacts influence cell cycle regulation, an aspect that so far has been unexplored.

**FIGURE 1:**
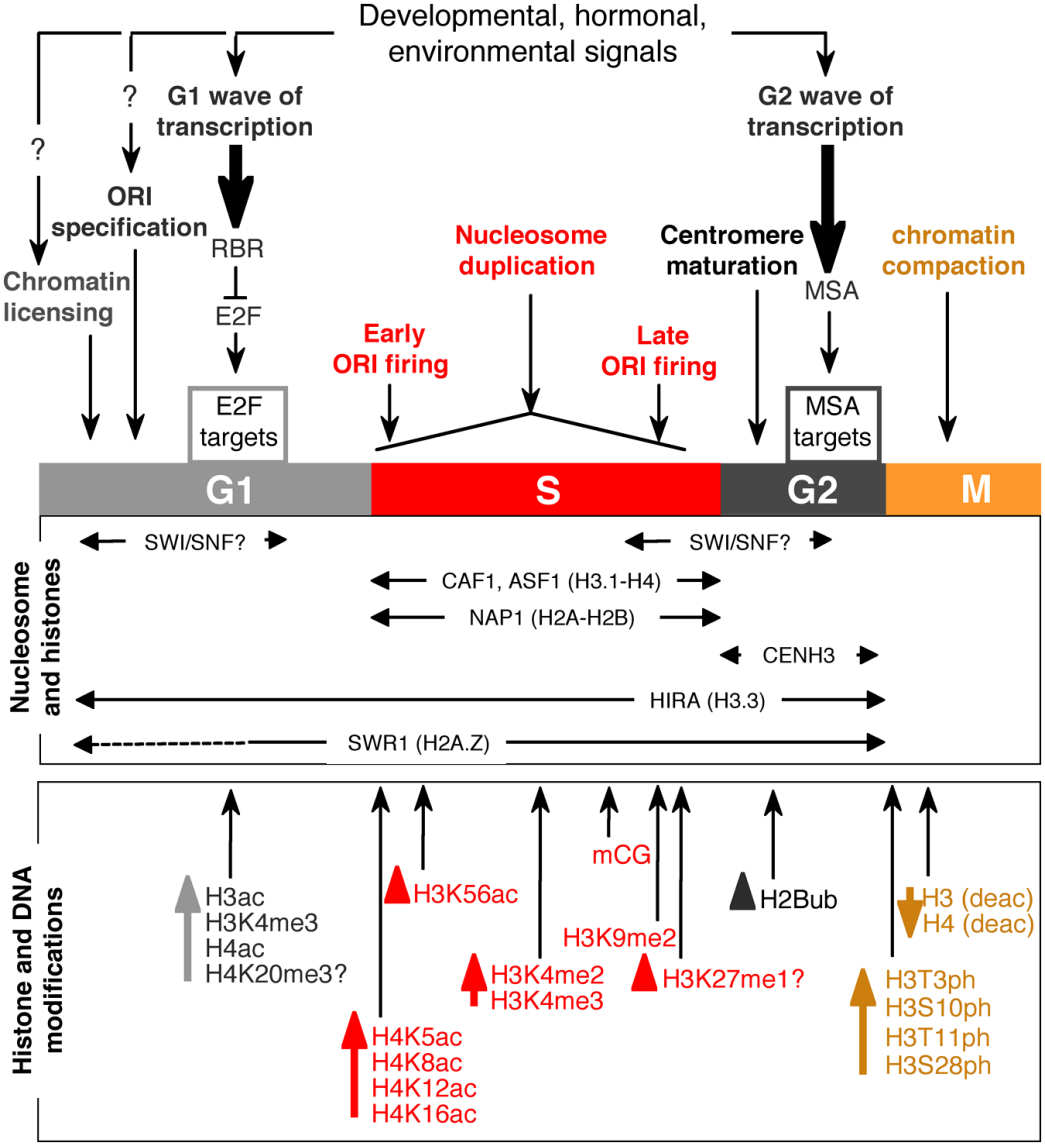
**Schematic view of chromatin processes intimately coordinated with cell cycle progression.** Changes in chromatin accessibility, which in many cases depend on histone modifications, histone exchange, and nucleosome reorganization, and transcriptional waves are depicted. Histone modifications are color-coded according to the cell cycle phase. Arrows in front of histone modifications denote changes associated with the corresponding cell cycle stage.

Therefore, the interface between cell cycle and exit to differentiation, with or without endoreplication events (De Veylder et al., 2007; Edgar et al., 2014), and chromatin dynamics can be summarized in the following processes: cell cycle-dependent transcriptional waves, genome duplication, and genome compaction and segregation (**Figure 1**). Thus, in this review we will focus on the accessibility of TFs to their targets and how chromatin modification enzymes and histone chaperones may affect transcriptional control during the cell cycle. Also, we will discuss aspects of genome duplication with an epigenetic perspective, that is, the role of chromatin status and modifications on replication factor binding, specification of replication origins, regulation of replication timing, prevention of re-replication, and the role of DNA replication factors in gene silencing.

## CHROMATIN LICENSING (EARLY G1)

One of the earliest events in the cell cycle is chromatin licensing, which is the process that allows various proteins and complexes to get access to chromatin. These include primarily DNA replication factors and chromatin modification proteins related to cell fate decisions.

Genome replication in eukaryotes requires the activation of thousands of replication origins, which are the genomic locations where initiation complexes bind to DNA and initiate DNA replication. This is a primary regulatory stage and the first event, that already started shortly after the two newly formed nuclei separate in late telophase, is the association of the pre-replication complex (pre-RC) at each of all potential replication origins. This is known as licensing and relies on a local increase of chromatin accessibility at potential origins (Sanchez et al., 2012; MacAlpine and Almouzni, 2013). Once formed, licensed origins contain the heterohexameric ORC, CDC6, CDT1, and the heterohexameric MCM2-7 complex. However, pre-RCs are not formed at random locations but rather there are sites that show a preference for pre-RC assembly (Karnani et al., 2010; MacAlpine et al., 2010; Costas et al., 2011a). The mechanism of origin specification is far from being understood (Mechali, 2010; Costas et al., 2011b; Sanchez et al., 2012; MacAlpine and Almouzni, 2013; Mojardin et al., 2013). In fact, purified mammalian ORC binds DNA *in vitro* with no sequence specificity (Vashee et al., 2001; Remus et al., 2004; On et al., 2014). This suggests that in higher eukaryotes, both animals and plants, the local chromatin environment is a primary determinant of pre-RC formation. More specifically, loading of the replicative helicase MCM in G1 in human cells seems to be affected by Hbo1, a histone acetylase that interacts and acetylates Orc2, Cdc6, and Mcm2 *in vitro* (Iizuka and Stillman, 1999; Burke et al., 2001; Iizuka et al., 2006; Miotto and Struhl, 2010). *Arabidopsis* contains two Hbo1 homologs, the HAM1 and HAM2 acetylases of the MYST family that may play a similar role in specifying pre-RC binding sites and/or stabilizing the complex.

Due to the developmental strategy and body organization of plants, organogenesis and cell differentiation, including cell fate decisions in response to developmental cues, must be highly coordinated with cell proliferation and growth (Fletcher, 2002; Gutierrez, 2005; De Veylder et al., 2007; Scheres, 2007). The pre-RC component CDT1 takes relevance here since in *Arabidopsis* it seems to be a multifunctional factor. It stimulates endoreplication in cells genetically programmed to undergo differentiation-associated endocycles and cell division in cells with certain stem cell potential (Castellano et al., 2004). In addition, it is also known to increase the expression of *GL2* (*GLABRA2*; Caro et al., 2007), a homeobox gene crucial for cell fate specification of atrichoblasts in the root epidermis (Schiefelbein et al., 2014). Chromatin at the *GL2* locus changes in a cell cycle-dependent manner. Thus, fluorescence in situ hybridization (FISH) experiments have demonstrated that a positive FISH signal is detected as early as in anaphase in epidermal cells at the *GL2* locus and it is soon afterward in early G1 that epidermal cell fate is decided (Costa and Shaw, 2006): chromatin remains highly accessible in atrichoblasts and consequently *GL2* is expressed while it becomes much less accessible in trichoblasts where *GL2* expression is shut off. Therefore, the activity of a pre-RC component, e.g., CDT1, in DNA chromatin licensing appears to coincide in time with cell fate decisions. Although preliminary data suggest that changes in H3 acetylation and H3K9 tri- and dimethylation occur in a cell cycle dependent manner at the *GL2* locus (Caro et al., 2007), further experiments are needed to determine in detail the changes in chromatin accessibility and histone modifications associated with the process of cell fate decision in the case of root epidermal cells as well as in other cellular settings.

## THE G1 TRANSCRIPTIONAL WAVE (MID G1)

### E2F-DEPENDENT TRANSCRIPTION OF CHROMATIN GENES

A characteristic feature of plant cells is that transcriptional control is of primary relevance in regulating the availability of cell cycle proteins and, in general terms, of proteins that are required in a cyclic manner during the cell cycle. Typically, the G1 transcriptional wave depends on the activity of the Rb/E2F module (Gutierrez et al., 2002; Berckmans and De Veylder, 2009), which in *Arabidopsis* consists of the RETINOBLASTOMA-RELATED (RBR) protein and various RBR-interacting E2F proteins, the so-called typical E2F (A, B, and C; Ramirez-Parra et al., 2007; Desvoyes et al., 2014; Kuwabara and Gruissem, 2014; **Figure 1**). The burst in E2F-mediated gene expression occurs only after the repressive action of RBR is abolished by phosphorylation of several residues in this protein that provokes its release from E2F complexes at the target promoters. Genome-wide data are now available from asynchronous and synchronous cell cultures that constitute a valuable resource to study E2F target genes expression (Menges et al., 2002, 2003, 2005; Ramirez-Parra et al., 2003; Vandepoele et al., 2005; Naouar et al., 2009). The presence of RBR favors the recruitment of various chromatin modification enzymes, such as histone deacetylases (HDAC), histone methyltransferases (HMTases), and DNA methyltransferases (Dnmt; Zhang and Dean, 2001; Macaluso et al., 2006).

In mammalian cells, expression of E2F target genes correlates with an increase in certain histone modifications such as H3K4me3 and H3ac (Takahashi et al., 2000; Taubert et al., 2004). Also, some of the chromatin modification enzymes associated with the G1 progression are themselves E2F targets or cell cycle regulated by other factors, thus acting as a positive regulatory loop. Among these, the MET1 (Vlieghe et al., 2003) and CMT3 Dnmt are examples of E2F-mediated gene expression, which are required at a later stage in the cell cycle since MET1 acts in coordination with DNA replication and CMT3 is a maintenance methylase. *MET1* expression is up-regulated in plants overexpressing E2Fa (Vlieghe et al., 2003) and repressed by RBR in cooperation with MSI1, clearly demonstrated in the female gametophyte central cell where it is required for proper heterochromatin maintenance (Johnston et al., 2008; Jullien et al., 2008). Regarding *CMT3*, data available from synchronized cells show an increase of expression coinciding with the G1/S transition (Menges et al., 2003). However, it is worth noting that CMT3 may have a role later in the cell cycle since its mRNA accumulation is maximal in late G2 (Sanchez et al., 2008), although expression of the de novo Dnmt DRM2 is not cell cycle regulated (Law and Jacobsen, 2010).

Other genes that are up-regulated in G1, and in a large proportion through the RBR/E2F pathway, are those encoding proteins required for genome duplication in S-phase such as all CDC6, CDT1, MCM3 and all pre-RC factors, except ORC5 (Castellano et al., 2001, 2004; Stevens et al., 2002; Diaz-Trivino et al., 2005), the large subunit of chromatin assembly factor CAF-1, FAS1, that deposits histone H3.1–H4 dimers in a DNA replication-dependent manner (Ramirez-Parra and Gutierrez, 2007a), and the ASF1 H3 chaperone (Lario et al., 2013).

A remarkable observation in regard to TF availability is that binding sites for various TF frequently colocalize with transposable elements (TE). In animal cells this is the case for OCT4 and NANOG, Sox2, c-Myc, and CTCF, among others (Bourque et al., 2008; Kunarso et al., 2010; Lynch et al., 2011; Schmidt et al., 2012; Jacques et al., 2013) and recently for E2F in several *Brassicaceae*, including *Arabidopsis* (Henaff et al., 2014). Thus, up to 85% of the sequences that fit the E2F consensus sequence in *Arabidopsis* are amplified in TEs and ChIP experiments show that they bind E2Fa *in vivo*, indicating that the overall availability of E2F can be affected by E2F binding to TEs. These data suggest that TEs located in the proximity of gene promoters may directly participate in their expression level and those in other locations affect the effective nuclear concentration of E2F and its transcriptional network (Henaff et al., 2014).

### HISTONE MODIFICATIONS AND NUCLEOSOME REMODELING IN G1

Histone acetylation must be also properly coordinated with the G1 transcriptional wave. Accordingly, several histone acetylases (collectively named HATs) are cell cycle regulated and exhibit a burst of expression in mid G1 (Sanchez et al., 2008). This step is normally associated with an increase in histone deacetylation carried out by HDACs. Given the similarity between mammalian and plant RB proteins, it is likely that the RB-HDAC interaction that occurs in mammalian cells (Brehm et al., 1998; Magnaghi-Jaulin et al., 1998) by binding to E2F target promoters (Lai et al., 1999; Ferreira et al., 2001) also takes place in plants. RBR phosphorylation may abolish interaction with HDACs, favoring HAT activity that relieves gene repression (Rayman et al., 2002). Such balance has been demonstrated in several plant species (Ach et al., 1997; Nicolas et al., 2001; Rossi and Varotto, 2002; Rossi et al., 2003).

Nucleosome remodeling carried out by SWI/SNF complexes that change the location of nucleosomes relative to genomic elements, e.g., promoters, also affects gene expression of the G2 transcriptional wave. In mammalian cells, Brm and Brg1, members of the SWI/SNF family, interact with RB and control the timely expression of cyclin A and E before initiation of S-phase (Dunaief et al., 1994; Zhang et al., 2000). Although *Arabidopsis* contains several SWI/SNF complexes, an interaction between RBR and BRM has not been demonstrated. Since BRM is highly expressed in dividing cells (Farrona et al., 2004; Knizewski et al., 2008; Efroni et al., 2013), it is tempting to speculate that SWI/SNF complexes may affect the G1 transcriptional wave, perhaps through RBR interaction.

## GENOME REPLICATION EVENTS AND CHROMATIN MODIFICATIONS (S)

### IS SPECIFICATION OF REPLICATION ORIGIN UNDER EPIGENETIC CONTROL?

Initiation of genome replication marks the beginning of S-phase that lasts until the entire genome is duplicated. There are several processes required for proper initiation and completion of genome replication that, interestingly, have revealed an intimate relationship with chromatin-related events. These include primarily chromatin accessibility and likely nucleosome remodeling, changes in specific histone modifications, and the participation of histone chaperones. The function of these factors is crucial for replication timing, origin specification and activity, and the re-replication control that restricts initiation at replication origins to once and only once per cell cycle. This is not surprising since not only the DNA has to be replicated during S-phase but also chromatin, quite importantly all the DNA and histone modifications that are present before replication (Costas et al., 2011b; MacAlpine and Almouzni, 2013).

A relatively small proportion of all origins marked with bound pre-RC are actually activated at the G1/S transition. The features that determine origin activation are not known although it seems clear that a local chromatin landscape, in addition to DNA sequence characteristics, are involved (Costas et al., 2011b; Sanchez et al., 2012; Mechali et al., 2013). A genome-wide map of origins (the “originome”) is now available for *Arabidopsis* cultured cells (Costas et al., 2011a). This dataset revealed a negative correlation between origins and CG methylation as well as a positive correlation with histone modifications frequently associated with active genes, such as H3K4me2, H3K4me3, H3ac, and H4ac, coinciding with data obtained in animal cells (Cadoret et al., 2008; Sequeira-Mendes et al., 2009; Karnani et al., 2010). They also tend to be located in genomic places enriched for nucleosomes and the histone H2A.Z variant. This genome-wide data is fully consistent with previous results from immunofluorescence analysis. Thus, progression through S-phase is associated with an increase in H3K18ac, H4K5ac, H4K8ac, H4K12ac, and H4K16ac in various plant species (Jasencakova et al., 2001, 2003; Mayr et al., 2003). This appears to be a general feature since it has been found also in animal species, including Xenopus, *Drosophila*, and human cells (Danis et al., 2004; Hartl et al., 2007; Schwaiger et al., 2009). One of these marks is enriched in active origins (Costas et al., 2011a) and in early replicating chromatin regions of *Arabidopsis* chromosome 4 (Lee et al., 2010; see also below).

It seems clear that origins tend to be associated with genomic regions enriched for histone modifications and variants present in active genes. However, it is worth noting that also large genomic regions with a low or fully repressed nature are replicated in S-phase and therefore must contain origins, perhaps with a distinct signature. In an effort to define chromatin domains that can be characterized by specific epigenetic landscape we have recently identified 9 major chromatin states in the *Arabidopsis* genome based on different combinations of 16 chromatin features including DNA sequence elements, CG methylation, histone variants, and histone modifications (Sequeira-Mendes et al., 2014). These studies have also revealed the topographical relationship between different states, which are not randomly placed next to each other but instead they follow a pattern of relatively few chromatin motifs. This information is of primary relevance for future studies aimed at defining chromatin signatures that are associated with replication origins and other regulatory elements in the genome. In any case, the major challenge ahead is to determine whether the various histone modifications are a cause of replication activity or if they actually determine origin activation. The use of various genetic and genomic tools available for *Arabidopsis* should be instrumental for this purpose.

### H3K56 ACETYLATION AND REPLICATION TIMING

Not all origins fire at once at the beginning of S-phase. There is a strict control of the time of origin activation, whereby some genome regions replicate early while others replicate late. As in most systems studied, two waves of genome have been observed in *Arabidopsis*, the early and late waves where euchromatin and heterochroatin, respectively, are replicated (Lee et al., 2010). However, the mechanism controlling timing is not understood. Although various mechanisms have been proposed to control replication timing, including a stochastic model (Bechhoefer and Rhind, 2012), a plausible mechanism that cooperates with random timing control is the association with certain chromatin features. Supporting this view, H3K56ac is frequently associated with early replication in *Arabidopsis* (Lee et al., 2010), as it is the case in animal cells (Kaplan et al., 2008; Gondor and Ohlsson, 2009). Whether the presence of H3K56ac is determinant of early replication is not known since the replication pattern of cells lacking this histone modification has not been studied. However, it is worth noting that heterochromatin, which replicates late in S-phase, does not contain detectable amounts of this mark.

### HISTONE MODIFICATIONS CONTROL THE RE-REPLICATION AVOIDANCE MECHANISM

When a given origin initiates replication multiple times within the same S-phase, genomic regions around that origin become re-replicated, a cause of chromosomal aberrations in mitosis (Arias and Walter, 2007; Drury and Diﬄey, 2009; Costas et al., 2011b). Several mechanisms have evolved in eukaryotic cells to prevent the deleterious consequences of re-replication, including selective proteolysis of pre-RC components, changes in their subcellular localization, and inhibitors of pre-RC (Saha et al., 2006; Drury and Diﬄey, 2009; Havens and Walter, 2009; Ding and MacAlpine, 2010; Miotto and Struhl, 2010; Wong et al., 2010). In addition, novel mechanisms involving the presence of certain histone modifications at origins have been identified. In animal cells, pre-RC assembly at origins depends on the presence of H4K20me1, which levels are cell cycle regulated and very low in S-phase (Yang and Mizzen, 2009; Tardat et al., 2010). The amount of H4K20me1 depends on the coordinated action of the Set7 methylase and the PHF8 demethylase: Set7 is absent in S-phase because after origin firing it is degraded by a PCNA- and Cul4-Ddb1-dependent process in the proteasome (Oda et al., 2009; Liu et al., 2010; Tardat et al., 2010). The presence of any form of H4K20 methylation has been questioned in *Arabidopsis* (Zhang et al., 2007), although immunofluorescence results indicate that H4K20me1 is associated with chromocenters whereas H4K20me3 with euchromatin (Fuchs et al., 2006; Sanchez et al., 2008; Desvoyes et al., 2010), pointing to a potential role of H4K20me3 in origin function. Less speculative is the role of H3K27me1 in controlling re-replication in *Arabidopsis* heterochromatin. This has been demonstrated using mutant plants lacking the *ATXR5* and *ATXR6* genes encoding the Trithorax-related H3K27 monomethyltransferases that exhibit abnormal re-replication control of the heterochromatin domains (Jacob et al., 2009, 2010). Furthermore, decreased methylation of cytosines suppresses the phenotype of the double *atxr5, atxr6* mutant (Stroud et al., 2012a). In this context, the enrichment of H3.1 variant in heterochromatin is a crucial part of the mechanism since H3.1 methylation by ATXR5 is selective due to a sterical hindrance in ATXR5 by the presence of a threonine residue at position 31 in H3.3 (instead of alanine in H3.1; Jacob et al., 2014). Interestingly, the defects in heterochromatin condensation of the *atxr5, atxr6* mutant are enhanced by overexpression of KRP5, a CDK inhibitor that plays a role in endoreplication control and cell elongation (Jegu et al., 2013), suggesting a link between heterochromatin status and endocycle control (Edgar et al., 2014).

### HISTONE DYNAMICS DURING GENOME REPLICATION

Genome replication is intimately coordinated with chromatin duplication, a process that needs continuous deposition of histone octamers on the newly synthesized DNA. This step is catalyzed by histone chaperones such as NAP1 (and NAP1-related protein), which transfers H2A-H2B dimers (Galichet and Gruissem, 2006; Zhu et al., 2006), ASF1 that loads H3-H4 dimers onto HIRA and CAF-1 (Zhu et al., 2011), and CAF-1 that brings H3.1–H4 dimers (Polo and Almouzni, 2006; Das et al., 2010). In the latter case, it is important to note that CAF-1 is evolutionarily conserved (Ramirez-Parra and Gutierrez, 2007b). This implies that an active exchange of H3.1 for H3.3 must be carried out by the specific exchange HIRA chaperone in the genome locations where it is required (Tagami et al., 2004). This is important because H3.1 and H3.3 are preferentially enriched in repressed and active chromatin, respectively, both in animal and plant cells (Stroud et al., 2012b; Wollmann et al., 2012). In addition, proper incorporation of H3.1 and its maintenance is crucial for heterochromatin silencing (Kirik et al., 2006; Schonrock et al., 2006; Stroud et al., 2012a; Jacob et al., 2014). Correct CAF-1 activity is also required during male gametogenesis in *Arabidopsis* (Chen et al., 2008b). Although plants are more tolerant to defects in CAF-1 function than mammals, alteration in the H3.1/H3.3 balance seems to be highly deleterious for plant development, as revealed by the pleiotropic phenotype of *fas1*, *fas2*, and *msi1* mutants, encoding each of the three CAF-1 subunits (Kaya et al., 2001; Hennig et al., 2003; Ramirez-Parra and Gutierrez, 2007a). Thus, *fas1* mutants show increased homologous recombination, limited TE silencing, telomere shortening, and loss of 45S rDNA repeats (Endo et al., 2006; Kirik et al., 2006; Ono et al., 2006; Schonrock et al., 2006; Mozgova et al., 2010; Jaske et al., 2013). Likewise, *asf1a, b* double mutants exhibit a S-phase delay and up-regulation of checkpoint genes, such as *ATM*, *ATR*, and *PARP1* (Zhu et al., 2011). Together, these data indicate that the location of H3.1 across the genome is finely controlled and very important for growth and development.

A major issue that needs to be taken into consideration is that chromatin is disassembled while replication proceeds and then reassembled past each replication fork during the entire S-phase. This requires the restoring of post-translational modifications in the newly formed chromatin in order to maintain the epigenetic states (Probst et al., 2009). For example, most of newly synthesized and deposited H4 contain H4K5ac and H4K12ac (Sobel et al., 1995; Loyola et al., 2006), frequently associated with active chromatin, but clearly these marks are not maintained in the entire set of H4 molecules in replicated chromatin. It has been speculated that these modifications serve to mark the location of newly formed chromatin for further processing (MacAlpine and Almouzni, 2013). Another histone mark that is characteristic of newly synthesized histones is the acetylation of lysine 56 in the core domain of H3 (H3K56ac). In yeast, these new histones are incorporated during S phase, together with the maternal histones that are transferred to the new daughter DNA strands. The H3K56ac mark is then erased during G2/M by Hst3 and Hst4 HDACs (Celic et al., 2006; Maas et al., 2006). This modification has been associated with DNA replication-coupled nucleosome assembly in several eukaryotes (Han et al., 2007; Kaplan et al., 2008; Li et al., 2008) and also with DNA damage response and chromatin assembly following DNA repair (Masumoto et al., 2005; Chen et al., 2008a). As already mentioned, in *Arabidopsis*, H3K56ac levels strongly correlate with early replicating regions (Lee et al., 2010), suggesting an association with nascent DNA behind the replication forks. Likewise, newly deposited H3 is very poor in lysine methylation in mammalian cells (and likely also in other systems), again a situation that needs to be modified past the replication fork to restore the local H3 methylation pattern. A genomic region where these changes are particularly evident is heterochromatin, on which the normal low levels of H3ac and H4ac and high levels of H3 methylation and CG methylation need to be restored quickly after fork progression (MacAlpine and Almouzni, 2013).

## THE G2 TRANSCRIPTIONAL WAVE

The G2 phase has been traditionally considered a period of time where the cell with a duplicated genome (and other cellular components) prepares for mitosis. This relatively passive view is far from what actually occurs during G2 since various complex and crucial processes are actively regulated, including some chromatin-related events. Thus, G2 progression requires several specific events such as a new transcriptional wave to generate the gene products required mainly in mitosis, histone modifications necessary to mark G2/M targets, the triggering of the DNA damage checkpoint, and the deposition of the centromere-specific histone CENH3.

Microarray data of synchronized *Arabidopsis* cultured cells demonstrated that during G2 a characteristic and well-defined transcriptional wave occurs (Menges et al., 2005). Genes with a G2-specific expression have been identified to contain in their regulatory regions the so-called M specific activator (MSA) DNA binding motifs (Ito et al., 1998), such as several *CYCB1* genes, required for G2 progression, and *KNOLLE* (Haga et al., 2007; Berckmans and De Veylder, 2009), required for cell plate formation. In addition, it has been demonstrated that the *hub1-1* mutants show a longer G2 and a characteristic mis-expression of G2 marker genes (Fleury et al., 2007), such as various *CYCA*, *CYCB*, and *CDKB* genes. Interestingly, the *HUB1* gene encodes for a RING E3 ligase that mono-ubiquitinates H2B at residue K143 in plants (K123 in yeast and K120 in vertebrates). This function is similar to that of the yeast homolog of HUB1, which is a requisite to increase H3K4me3 (and concomitantly H3K36me3 and H3K79me3) in G2 expressed target genes (Xiao et al., 2005; Zhu et al., 2005). In addition, H2Bub is required for other cellular functions some of them with a likely relationship with cell proliferation, e.g., the balance between vegetative and reproductive development (Lolas et al., 2010), the circadian clock (Himanen et al., 2012), and photomorphogenesis (Bourbousse et al., 2012). In other cases such as in the regulation of flowering through FLC expression (Cao et al., 2008; Gu et al., 2009) or the plant immunity (Dhawan et al., 2009; Zou et al., 2014), a connection with cell proliferation is less evident.

Centromeres contain an atypical histone H3 both in sequence and size, called CENH3 in plants and CENP-A in animals which is a H3 variant quite different from other H3 proteins, such as the canonical H3.1 and the H3.3 variant (Muller and Almouzni, 2014; Otero et al., 2014). CENH3 is present in different plant species (Nagaki et al., 2012) and in the case of *Arabidopsis* it is encoded by the *HTR12* gene (Talbert et al., 2002), which is an E2F target gene with a peak of expression in mid-late G2 (Heckmann et al., 2011). In animal cells CENH3 deposition depends on the HJURP chaperone and it largely occurs in G1, where HJURP-CENH3-H4 complexes are active (reviewed in Muller and Almouzni, 2014). The timing of CENH3 incorporation in plants seems conserved in dicot and monocot plants (Nagaki and Murata, 2005; Lermontova et al., 2007) but it differs considerably from animal cells. Experiments using fluorescently tagged CENH3 have demonstrated that in *Arabidopsis* deposition occurs in late G2 and it does not depend on the centromeric DNA repeats (Heun et al., 2006; Mendiburo et al., 2011; Teo et al., 2013). While a HJURP homologue has not been identified in plants, other members of the pathway, such as Mis18 binding protein 1 (De Rop et al., 2012), have been recently identified in *Arabidopsis* as the *KINETOCHORE NULL 2* (*KNL2*) gene product (Lermontova et al., 2013). Interestingly, *KNL2* is also an E2F target expressed in G2 (Lermontova et al., 2013), reinforcing the importance of E2F-dependent transcriptional waves both in G1 and G2.

## MITOSIS

Mitosis marks the phase where newly formed chromosomes are segregated to the daughter cells. From the chromatin perspective, enormous changes are required for correct chromatin compaction, a process that is necessary to convert the relaxed and expanded genetic material present in interphase in compact chromosomal units that can be managed for segregation during mitosis. The N-terminal tail of H3 is the location where, at least, four major phosphorylations occur: H3T3ph, H3S10ph, H3T11ph, and H3S28ph. This set of phosphorylation events is largely conserved in animals and plants, although the pattern in meiosis differs (Manzanero et al., 2000; Houben et al., 2007; Rossetto et al., 2012). H3 phosphorylation at threonine (T) residues appears to be specific for mitotic compaction whereas phosphorylation at serine (S) residues also occurs in meiosis (Houben et al., 1999, 2005; Kaszas and Cande, 2000; Manzanero et al., 2000). H3T11ph, which in animal cells is predominant in centromeric regions, is present along the chromosomes in plants (Houben et al., 2007). Phosphorylation is not exclusive of canonical H3 since it is also detectable in CENH3 where it serves to demarcate the boundaries of pericentromeric chromatin (Zhang et al., 2005). In human cells, H3 phosphorylation is associated with chromatin compaction in mitosis and is accompanied by a generalized shut-down of transcription and a decrease in histone acetylation, not only at residues H3K18 and H3K23 but also in H4 (residues K5, K8, K12, and K16; Bonenfant et al., 2007). Detailed studies in this direction are not available in plants.

A plethora of kinases present in human cells are known to use all histones as substrates, e.g., more than 15 kinases phosphorylate different residues of H3 (Rossetto et al., 2012). Among them some are present in plant cells and it is conceivable that they play a similar role, the Aurora kinases being major players in histone H3 phosphorylation. *Arabidopsis* contains three *AUR* genes that have a characteristic expression pattern depending on the kinase (Demidov et al., 2005; Kawabe et al., 2005). The α-type AUR1 and AUR2 accumulate in the nuclear membrane in interphase and in the mitotic spindle during mitosis whereas the β-type AUR3 is uniformly associated with chromatin in anaphase (Kawabe et al., 2005). This pattern coincides with the level of H3S10ph in mitosis (Demidov et al., 2005; Kawabe et al., 2005). Similar conclusions are derived from studies in tobacco cells (Kurihara et al., 2006). *In vitro* experiments have served to determine that AUR1 specifically phosphorylates histone H3 at S10 but neither at S28 nor at T3 and T11. The latter two phosphorylation sites are the substrate of Haspin, another mitotic kinase required for the accumulation of AUR3 at centromeres in metaphase (Kurihara et al., 2011). Interestingly, AUR1 activity on H3S10 is facilitated by H3K9ac and inhibited by H3K14ac, while H3S10ph interferes with H3K9me2, revealing a complex crosstalk between different H3 modifications (Demidov et al., 2009). At the organismal level, AUR1 and AUR2 activities play a role in formative cell divisions during organogenesis as revealed by the severe phenotype of *aur1* and *aur2* mutants related with positioning the cell plate (Van Damme et al., 2011) and establishing the primary root meristem (Petrovska et al., 2012).

## CHROMATIN DYNAMICS DURING THE MEIOTIC CELL CYCLE

Meiosis is a complex process, highly conserved in eukaryotes and crucial for sexual reproduction since it ends up with the production of gametes. In this highly specialized cell cycle, two successive events of chromosome segregation occur in the absence of any intervening genome replication, thereby leading to the reduction in the ploidy level and the production of haploid gametes. The prophase of the first meiotic division, a long, structurally and functionally complex stage, is when recombination events take place. The location of meiotic crossovers (CO) hot spots is epigenetically determined. They are enriched in marks associated with open chromatin, such as H2AZ and H3K4me3, and present low level of DNA methylation (Choi et al., 2013). Moreover, *met1* mutant, present an anormal increase of CO in the pericentromeric regions (Yelina et al., 2012). Less considered, it is the interphase period prior to entering meiosis, where a S-phase, originally identified in *Liliaceae* (Taylor and McMaster, 1954), occurs and that in *Arabidopsis* has been shown to be longer than in the mitotic cell cycle and with the eu-and heterochromatin uncoupled (She et al., 2013). In *A. thaliana*, the entire meiosis, spanning from the premeiotic S-phase to tetrad production, takes ~36 h (Armstrong and Jones, 2003). During this period changes at the chromatin level occur, both in terms of chromosome condensation and histone modifications, which have been primarily revealed by immunofluorescence microscopy (reviewed in She and Baroux, 2014).

Given the significant condensation and decondensation events characteristic of meiosis, it is not surprising that the histone H3S10ph shows a cyclic labeling pattern in meiotic chromatin. Thus, H3S10ph-positive chromatin is first detectable in diplotene and chromosomes remain strongly and uniformly labeled until anaphase I is finished. Then the labeling disappears until initiation of the second meiotic division, when the H3S10ph signal again becomes apparent (Oliver et al., 2013). A largely similar pattern is also observed in various cereal species (Manzanero et al., 2000). Other histone modifications associated with active chromatin (H3K9K14ac, H3K4me2/me3), heterochromatin (H3K9me2), and Polycomb chromatin (H3K27me3) do not exhibit very significant changes in pollen mother cells (Oliver et al., 2013). However, the situation is different in megaspore mother cells where reprogramming involves depletion of H1 linker histones and changes in histone variants and post-translational modifications (see She and Baroux, 2014; for a detailed discussion). It is worth noting that H3K9K14ac appears rather constantly through different meiotic stages in spite of that histone deacetylation has been associated with chromosomal packaging (Xu et al., 2009). The only differences observed between dicotyledonous and monocotyledonous plants are derived from the distinct chromosomal location of certain chromatin regions, e.g., H3K9me2 and repetitive sequences.

## CONCLUDING REMARKS

The relevance of chromatin for cell cycle regulation has been highlighted based on accumulating evidence that significant chromatin modifications are associated with cell cycle events (Sanchez et al., 2008). A major question is whether these modifications trigger specific cell cycle events or are required for specific cell cycle transitions. There are a few examples, reviewed in this article, supporting the idea that this seems to be the case. The current available information points to the existence of, at least, several cell cycle events intimately linked to and/or dependent on specific chromatin changes such as, replication origin licensing, G1-specific gene expression, replication origin specification and activation, chromatin replication, centromere maturation, G2-specific gene expression, and chromatin compaction. However, the number of cell cycle processes with a direct relationship with DNA and chromatin dynamics is increasing as new lines of evidence emerge. The better knowledge that is being acquired on the enzymatic activities that modify chromatin will be crucial in the near future to delineate the mechanisms of chromatin-mediated cell cycle progression. Thus, analysis of cell cycle kinetics under conditions where chromatin functions are impaired should illuminate the field. In this context, research in plant systems should contribute very positively to the advancement in the chromatin basis of cell cycle control since a large amount of mutants are available with known defects in chromatin-related enzymatic activities. Furthermore, given the significant growth plasticity of plants bearing mutations in key genes, it would be possible to analyze cell cycle regulation during organogenesis, an aspect that is far more complex to approach in animal models.

## Conflict of Interest Statement

The authors declare that the research was conducted in the absence of any commercial or financial relationships that could be construed as a potential conflict of interest.
